# Improving Palliative Care for Cancer

**DOI:** 10.1038/sj.bjc.6600646

**Published:** 2002-12-02

**Authors:** Adrian Tookman

**Affiliations:** Royal Free Hospital, London, UK

## Abstract

*British Journal of Cancer* (2002) **87**, 1481–1481. doi:10.1038/sj.bjc.6600646 www.bjcancer.com

© 2002 Cancer Research UK

## 

‘Improving Palliative Care for Cancer’ is an independent review that gives a fascinating overview of palliative care in the United States. Prepared in accordance with procedures approved by the National Research Council's Report Review Committee it has been reviewed by a panel of American experts and offers an opportunity to learn of the services, problems and aspirations of a rapidly expanding area of cancer medicine. This book is a report that ‘defines the major barriers that keep people from receiving excellent palliative care, as needed, throughout the course of their illness with cancer and recommends a series of steps forward.’ Throughout this report (it is a ‘report’ that is 300 pages in length) one can not help but make comparisons between palliative care in the US and in the UK. The problems presented to Palliative Care Services by the economic environment imposed by the American health care system are very different to problems faced by palliative care services in the UK. There are lessons to be learnt from a reimbursement system that appears to disadvantage patients who have palliative care needs. For example, there are obvious shortcomings presented by a reimbursement system that will only support patients in a palliative care programme for 6 months, which focuses palliative care on end of life issues. This limited approach to care will be alien to specialists in the UK where many palliative care services work in partnership with oncology services, supporting patients through treatment as well as in the terminal phase of illness.

The report is divided into two parts. The first part is an executive summary with background information, barriers to excellent palliative care and end of life care, conclusions and recommendations. One key recommendation is to designate cancer centres as centres of excellence in symptom control and palliative care which can become model services with high quality research and education. This theme has a familiar sound to those of us in the UK where cancer centres are now established. However, the mechanisms by which this high quality, evidence based care are to be disseminated are not detailed. In the UK we have attempted to address inequity in cancer, palliative care and supportive care by establishing Clinically Managed Networks. I felt there needed to be an attempt to discuss this important issue in more detail in this report. Other recommendations include developing multi professional guidelines, developing information services and ensuring that there is adequate palliative care representation on the National Cancer Institutes’ advisory bodies. In addition it is recommended that private insurers should provide adequate compensation for end-of-life care.

The second part comprises eight commissioned papers on key areas in palliative care in the United States. There is a common format to these. The current ‘state of play’ is presented with well-referenced discussions that are understandably focused on American literature. The problems are then presented and then there is debate about future directions. Finally, conclusions and recommendations are drawn. The contents of these papers are mixed and in places open to criticism. There is a noticeable lack of information on bereavement services, a review of symptom control that lacked depth, and a polarised view of palliative care research. However, for the informed reader, this report would form the basis for discussion and debate about many key issues in contemporary palliative care for patients with cancer. I personally found some of the recommendations repetitive and lacking focus or clear direction. My concern was that they could potentially be ‘wish lists’ that had little prospect of being implemented. In addition, I can't help but feel that there will need to be a huge specialist and bureaucratic resource to carry out all these ambitious recommendations. However, if they are to be supported and funded then this could certainly place palliative care in the USA in a world-leading position. One can only wish Palliative Care in America good luck in its task.

Improving Palliative Care for Cancer is a major review of palliative care in the US in its totality. There has never been a similar review in the UK. Our services have evolved in response to the changing needs of cancer care. Perhaps the continuing success of the speciality in the UK is a reflection of this approach. Evolution or revolution? Read the report to decide – there is much food for thought.

